# Moderated Online Social Therapy for Carers of Early Psychosis Clients in Real-World Settings: Cluster Randomized Controlled Trial

**DOI:** 10.2196/47722

**Published:** 2023-09-06

**Authors:** John Gleeson, Ashleigh Lin, Peter Koval, Liza Hopkins, Paul Denborough, Reeva Lederman, Helen Herrman, Sarah Bendall, Dina Eleftheriadis, Sue Cotton, Yael Perry, Michael Kaess, Mario Alvarez-Jimenez

**Affiliations:** 1 Healthy Brain and Mind Research Centre School of Behavioural and Health Sciences Australian Catholic University Fitzroy Australia; 2 University of Western Australia Nedlands Australia; 3 Melbourne School of Psychological Sciences The University of Melbourne Parkville Australia; 4 Alfred Health Melbourne Australia; 5 School of Computing and Information Systems Faculty of Engineering and Information Technology The University of Melbourne Parkville Australia; 6 Orygen Parkville Australia; 7 Centre for Youth Mental Health The University of Melbourne Parkville Australia; 8 University Hospital of Child and Adolescent Psychiatry and Psychotherapy University of Bern Bern Switzerland

**Keywords:** first-episode psychosis, carers, eHealth, families, stress, psychosis, digital mental health intervention, web-based therapy, social therapy

## Abstract

**Background:**

Family carers of youth recovering from early psychosis experience significant stress; however, access to effective family interventions is poor. Digital interventions provide a promising solution.

**Objective:**

Our objective was to evaluate across multiple Australian early psychosis services the effectiveness of a novel, web-based early psychosis intervention for carers.

**Methods:**

In this cluster randomized controlled trial conducted across multiple Australian early psychosis services, our digital moderated online social therapy for carers (Altitudes) plus enhanced family treatment as usual (TAU) was compared with TAU alone on the primary outcome of perceived stress and secondary outcomes including mental health symptoms and family variables at the 6-month follow-up.

**Results:**

Eighty-six caregivers were randomized and data were available for 74 young people in their care. Our primary hypothesis that carers randomized to Altitudes+TAU would report greater improvements in perceived stress at follow-up compared with carers randomized to TAU alone was not supported, with the TAU alone group showing more improvement. For secondary outcomes, the TAU alone group showed improved mindfulness over time. Regardless of group assignment, we observed improvements in satisfaction with life, quality of life, emotional overinvolvement, and burden of care. In contrast, hair cortisol concentration increased. Post hoc analyses revealed more contact with early psychosis services in the intervention group compared to TAU alone and that improvements in perceived stress and social support were associated with use of the intervention in the Altitudes+TAU group. In this study, 80% (12/15) reported a positive experience with Altitudes and 93% (14/15) would recommend it to others.

**Conclusions:**

Our trial did not show a treatment effect for Altitudes in perceived stress. However, our post hoc analysis indicated that the amount of use of Altitudes related to improvements in stress and social support. Additional design work is indicated to continue users’ engagement and to significantly improve outcomes in problem-solving, communication, and self-care.

**Trial Registration:**

Australian and New Zealand Clinical Trials Registry ACTRN12617000942358; https://trialsearch.who.int/Trial2.aspx?TrialID=ACTRN12617000942358

## Introduction

Psychotic disorders most often develop during late adolescence. In most cases acute positive psychotic symptoms, namely, hallucinations, delusions, and thought disorder, are responsive to treatment [1]. However, the recovery phase is often accompanied by deficits in psychosocial functioning (ie, lack of engagement with social relationships and vocational or educational pathways) [2] and secondary mental health symptoms [3-5]. In more than 50% of cases, psychotic relapse occurs during the first 3 years after onset of a first episode of a psychotic disorder [6].

Formal treatments for early psychosis are provided within treatment systems that prioritize early detection and comprehensive biopsychosocial interventions, with antipsychotic medication as the cornerstone [7]. Psychosocial interventions focus on recovery of psychosocial functioning via cognitive behavior therapy, vocational rehabilitation, and cognitive remediation [7]. Given the age of onset, families are the mainstay of informal care in early psychosis. A warm family environment is an important protective factor during recovery [8]; however, the burden of caring for a family member with early psychosis is associated with elevated distress [9].

As highlighted by the World Health Organization (WHO), the impacts upon caregivers warrant the dissemination of these effective family interventions for the benefit of both carers and the young person experiencing psychosis [10]. Meta-analytic evidence supports the effectiveness of family interventions in reducing relapse rates in early psychosis as compared to treatment as usual (TAU) [11]. However, access to family interventions is poor [10], resulting in missed opportunities for prevention [12]. This study is aimed at addressing poor accessibility via the provision of a family intervention using digital technology.

Digital mental health interventions provide a promising solution for poor accessibility [13]. Developed in partnership with carers and based on our Moderated Online Social Therapy (MOST) framework [14-17], our Altitudes intervention integrates evidence-based psychoeducation, peer-to-peer social networking, and web-based moderation in a single digital application [18].

We recently evaluated Altitudes via a cluster randomized controlled trial (ACTRN12616000968471). We compared Altitudes plus specialist first-episode family TAU with specialist first-episode family TAU alone within a single flagship early psychosis program [19,20]. At the 6-month follow-up, carers in both groups significantly improved on the primary outcome of stress in addition to a range of secondary outcomes, including mental health symptoms, carer self-efficacy, and expressed emotion [20]. In addition, there were significantly fewer visits to emergency departments by patients with early psychosis from the Altitudes group [20]. However, the trial was conducted at the Early Psychosis Prevention and Intervention Centre—a long-established, flagship early psychosis program in Melbourne, Australia, embedded in an academic research program that may have resulted in enriched background treatments [21]. This left open the question of whether Altitudes could add significant benefit for carers attending real-world early psychosis programs. Testing the feasibility of providing Altitudes across multiple early psychosis sites based across different cities was also a critical additional step in assessing its scalability.

Our aim in this trial was to evaluate whether a digital intervention (Altitudes), which uses MOST, improved perceived stress at the 6-month follow-up in carers with a relative receiving treatment for early psychosis, when added to real-world early psychosis services (ACTRN12617000942358).

The primary hypothesis was that carers randomized to the Altitudes web-based application+enhanced family TAU would report significantly greater improvements in perceived stress at the 6-month follow-up compared with carers randomized to enhanced TAU alone. The secondary hypothesis was that carers randomized to Altitudes+TAU would experience reduced activity of the hypothalamic-pituitary adrenal (HPA) axis—one of the major stress response systems of the human body—as well as improved positive coping, self-efficacy, depression, and perceived social support compared with carers randomized to TAU alone at 6 months follow-up.

## Methods

### Design

The East-West Altitudes trial was a single-blinded, cluster randomized controlled trial with clusters comprising individual families. A cluster design, with family as the unit of randomization, was selected to ensure that members of the same family were randomized to the same condition. It would have been infeasible to have family members participating concurrently in different treatment groups. The 2 treatment conditions included Altitudes plus enhanced treatment as usual (TAU) and enhanced TAU alone. The assessment time points were baseline (prior to randomization), 3 months (for the primary outcome only), and 6 months. The clinical sites were in Melbourne and Perth on Australia’s southeast and west coasts, respectively.

### Recruitment

Recruitment of the trial participants was undertaken at 13 sites across Melbourne and Perth between October 2018 and October 2019 including 1 clinical service in Melbourne (headspace early psychosis; hEP) and 2 in Perth (hEP and Western Australia Department of Health Early Intervention in Psychosis Services). Follow-up was completed in July 2020. The Melbourne hEP was managed by Alfred Health and consisted of a hub and spokes model across 5 sites (Bentleigh [hub], Elsternwick, Frankston, Dandenong, and Narre Warren). The Perth hEP consisted of the hub and spoke model of Joondalup, Osbourne Park, and Midland. The Western Australia Department of Health Early Intervention in Psychosis Services included coordinated sites, namely, Bentley, Fremantle, Peel, Rockingham, and Kwinana.

The research assistant (RA) met with potential participants face-to-face at the early psychosis service or in a location convenient to the carer. The RA presented the information regarding the study in writing and orally and invited prospective participants to ask any questions about any aspect of the study before obtaining signed informed consent.

### Ethics Approval

Ethics approval for Victorian sites was granted by the Alfred Hospital Human Research Ethics Committee (project No 298/17) and by Western Australia Department of Health (PRN: RGS0000000416) and University of Western Australia (RA/4/20/4245) for Western Australian sites.

### Participants and Setting

Eligible participants included carers who were aged ≥18 years (namely parents, grandparents, siblings, and partners) of young people who were currently receiving treatment at an early psychosis service (n=86). Up to 4 family members were eligible to participate from each family. For hEP services, eligibility criteria for the clients were (1) age 12-25 years; (2) a diagnosis of a first episode of psychotic disorder or at ultrahigh risk for developing psychosis [22]; and (3) no more than 12 months of continuous care upon presentation. For Western Australia Health, early psychosis services eligibility criteria for clients were (1) age between 16 and 40 years; (2) the client presented with early psychosis (no diagnosis required); (3) no more than 12 months of continual care upon presentation; and (4) duration of untreated psychosis ≤3 years.

Carers who did not have sufficient English to provide informed consent were excluded. We determined our target sample size based on an a priori power analysis, for which we assumed a moderate treatment effect (Cohen *d*=0.5) for our primary outcome of perceived stress at the 6-month follow-up [23]. Setting α at .05 (2-tailed), we determined that a sample size of 64 per group was required to achieve 80% power (G*Power Release; version 3.1.9.2; Heinrich Heine Univeritat Dusseldorf). Adjusting for the design effect (equal to 1.05), this equated to 68 per group or a total of 136 participants.

### Interventions

#### Overview

The Altitudes+TAU condition involved participation in our digital application [18]. TAU comprised the usual array of services for carers at each service in addition to a psychoeducation booklet. Altitudes, powered by our MOST software framework [14], integrated within 1 web-based application: evidence-based psychoeducation, peer-to-peer social networking, and expert and peer web-based moderation. Each user could log on 24 hours per day for the duration of the trial.

#### Altitudes Interactive Psychoeducation

The web-based psychoeducation was developed to target carer stress. New users were given an introductory welcome to Altitudes which highlighted ways to optimize their use of the system and how to access system help. Users were invited to complete, at their own convenience, a series of 8 web-based modules (known as “pathways”) which addressed themes of self-care, understanding psychosis, early warning signs and prevention of relapse, understanding their personal strengths as a carer, communicating with their relative, dealing with unhelpful thinking, self-compassion, and mindfulness. These pathways were divided into thematically-related psychoeducation “steps” to maximize the usability of the material. Each step was designed to be completed within 5-20 minutes (see Multimedia Appendix 1 for details for each step). The content of these steps comprised text, illustrations, and audio tracks and were designed to improve carer stress, for example, by encouraging self-care and by targeting problematic appraisals known to increase carer stress. In addition, the content of steps was influenced by social cognition concepts of “agency” and self-efficacy in family life [24]. The steps and pathways entailed regular prompts to users to share their reactions to material with other users through a series of “talking points.” Users’ responses populated the content of the social networking newsfeed. To facilitate the process of reflection on the content by participants, moderators could also contribute to the talking points. In addition, users could indicate their preference for material through “like” buttons, share content with other users, and keep track of which users had completed specific pathways and which users shared their specific personal strengths.

#### Altitudes Social Networking Features

The Altitudes social network enabled users to develop a web-based profile, communicate via posts with other users and web-based moderators, and comment on the web-based psychoeducation material. The application was hosted on a secure University of Melbourne web server. In addition, the web application included measures to secure the application and database against unauthorized access. Privacy and web-based safety were managed in accordance with the Online Social Networking Guidelines published by Cybersmart, a national cybersafety and cybersecurity education program managed by the Australian Communications and Media Authority.

#### Role of Moderators

In contrast to web-based self-help without human support, the guidance provided by moderators was integral to Altitudes. The model of web-based moderation was informed by the Supportive Accountability Framework of eHealth interventions, which emphasizes the importance of human support for engagement in eHealth systems [25].

Expert moderators were clinical psychologists with specialist family work experience. Their role was to optimize engagement, suggest specific content, facilitate joint problem-solving, and monitor safety daily. If the engagement was low, moderators would prompt participants via follow-up phone calls.

Peer moderators, with lived experience of caring for a relative with psychosis, modeled the use of the system and facilitated web-based interactions. At weekly supervision sessions with author JFMG, moderators reviewed progress and system engagement.

#### Enhanced Family TAU

All participating carers received a psychoeducation booklet that included information about psychosis and treatment, helpful contacts, as well as coping skills and suggestions for ways to assist and communicate with their relative. Additional carer services potentially included meetings with their relative’s case manager, psychiatrist, family peer worker, or carer consultant, as well as access to carer support groups. These differed by service.

### Measures

#### Primary Outcome

Perceived stress in carers over the preceding month was measured by the Perceived Stress Scale (PSS) [26]—a valid and reliable 10-item measure rated on a Likert scale ranging from 0 (never) to 4 (very often).

#### Secondary Outcomes

Hair cortisol is a biomarker of basal HPA axis activity [27]. The advantages of this measure are the validity as an index of long-term systemic cortisol levels, its reliability across repeated assessments, and its relative robustness to a range of potential confounding influences [28]. To assess potential changes of this biological stress response system, mean baseline HPA system activity during the last month was measured by a validated procedure for measuring hair cortisol [27]. Carer depressive symptoms were measured via the Centre for Epidemiological Studies Depression Scale—Revised [29]and substance use via the Alcohol, Smoking and Substance Involvement Screening Test [30]. Worry was measured via the Penn State Worry Questionnaire [31], loneliness via the UCLA (University of California, Los Angeles) Loneliness Scale [32], and social support via the Medical Outcomes Study Social Support Survey (MOS-SSS) [33]. The Me as a Parent Questionnaire was used to measure parental self-efficacy [34], coping was assessed via the Ways of Coping Scale [35,36], and personal strengths use via the Strengths Use Scale [37]. Self-compassion was measured via the Self-Compassion Scale Short Form [38] and mindfulness using the Mindful Attention Awareness Scale [39]. Satisfaction with life was assessed by the Satisfaction With Life Scale (SWLS) [40] and emotional, psychological, and social well-being via the Mental Health Continuum Short Form (MHC-SF) [41]. Quality of life (QoL) was measured by the Assessment of Quality of Life-8 dimensions [42].

#### Patient and Family Characteristics

Carer demographic variables included age, living situation, years of education completed, employment and marital status, country of birth, and source of income. Relevant family-level variables were measured including expressed emotion measured via the Family Questionnaire [43], and the degree of openness and extent of problems in family communication using the Parent-Adolescent Communication (PAC) scale [44]. Carer burden was assessed via the Experience of Care-giving Inventory [45]. The demographic characteristics of the young person were collected from carers.

A Resource Use Questionnaire was used to determine resource and treatment use by carers and young people. The use of services provided by the early psychosis service was documented via a self-report survey.

#### Altitude-Specific Measures

The use of Altitudes was continuously monitored across the study intervention period via frequency of log-ons. At 6 months, Altitudes users completed a self-report measure of their perception of Altitudes moderation [46] and a self-report usability measure [47,48].

### Procedure

RAs attended clinical team meetings across sites to promote the study. Participating carers were asked if their young relative could be approached to seek their consent to access data from their medical record. The young person was contacted by the study RA. The RAs undertaking the follow-up assessments were kept blind to treatment allocation. The participants were not blinded to their treatment allocation, and they were aware that Altitudes was the intervention of interest.

Randomization occurred after each baseline assessment. An independent statistician created the randomization sequence, which included permutated blocks. The block sizes and randomization sequence were concealed from the study coordinator, RAs, and investigators. The study coordinator randomized the family via a secure web-based clinical trials management system with stratification by state (Victoria or Western Australia). The system generated an email to the RA who telephoned the participant to let the family know which group they had been allocated to.

The primary and secondary outcomes were measured prior to randomization and repeated at the 6-month follow-up. The PSS was completed at 3 months using a telephone-administered version.

To enable analysis of hair cortisol, a single hair sample (at least 3 cm long, approximately 0.5 cm in diameter) was taken from a posterior vertex region on the head and stored at an ambient temperature. Screening prior to collection of the hair sample was used to determine factors that may affect the analysis of cortisol, such as hair products used. The hair cortisol analysis procedures included repeated washing of hair samples with isopropanol, drying, weighing (weight: 7.5, SD 0.5 mg), steroid extraction with methanol, evaporation of methanol (at 50 °C), and cortisol determination [27]. This analysis provided a total pg/mg value of cortisol in the hair corresponding to approximately one month prior to sampling.

### Statistical Analysis

Statistical analyses were conducted using Stata (version 16.1; StataCorp). Statistical analyses are presented in terms of the CONSORT (Consolidated Standards of Reporting Trials) extension pertaining to cluster analyses [49] and the International Conference on Harmonization Topic E9 Statistical Principals for Clinical Trials. Descriptive statistics were used to describe the total cohort as well as separately for the Altitudes+TAU and TAU alone groups. Baseline differences between the groups were not analyzed using inferential statistics as recommended in CONSORT guidelines [50]. Because of the randomization process, any baseline difference between 2 treatment groups is the result of chance and not due to external factors impacting treatment allocation [51]. Therefore, it is considered to be absurd to test for such differences and there have been calls for such practices to be eliminated [52]. Inferential statistics were used to compare baseline differences between caregivers who did and did not have follow-up data. For intent-to-treat analysis (ITT), all cases were included in the analyses, regardless of whether they had follow-up data.

For the primary outcome variable, the PSS, between group differences were examined using mixed effects repeated measures (MMRM) models [53]. In MMRM models, all observed information is used to derive the models (including estimation but not imputation of missing data). They are considered the preferred method for ITT analyses in clinical trials [54]. For these models, individual timepoint measures are considered nested within individual carers, who may be considered nested within families. For the main analysis, the parameters included group, time (includes baseline, 3 and 6 months), and the group x time interaction. All cases with at least 1 observation were included in the ITT. The default independence covariance structure was modeled in these models. As per protocol, analyses were also conducted with participants who had completed the intervention induction and had follow-up data. For resource use data, group differences were examined using either chi-square or Fisher exact test. Simple Pearson correlations were calculated for the association between parameters of intervention usage and outcomes in the Altitudes group.

## Results

### Participant Characteristics

There were 86 caregivers that participated in the study, with most being female and a mother of the young person (see Table 1). Caregivers ranged in age from 18 to 76 years (mean 51.2, SD 9.9 years). Most of the cohort were in married or de facto relationships and lived in their own house or flat. Just over 50% (n=46) of caregivers were born in Australia. Of those born overseas, 37% (n=15) were from European countries. Just over three-quarters of caregivers had completed their secondary school education and most had full-time employment. There were 46 caregivers from Victoria and 40 from Western Australia.

In the study, there were 74 young people. For 85% (n=63) of the young people, there was 1 caregiver in the study, 13% (n=10) had 2 caregivers and 1 young person had 3 caregivers. The mean cluster size was therefore 1.16 (SD 0.41). The characteristics of the young people are detailed in Table 2.

**Table 1:** Baseline demographic characteristics of the total cohort of caregivers (N=86) as well as separately for the Altitudes plus treatment as usual and only treatment as usual groups.

Variable			Total cohort (N=8)		Altitudes (n=43)		TAU^a^ (n=43)
Female (gender), n (%)			71 (83)		35 (81)		36 (84)
Age (years), mean (SD)			51.2 (9.9)		51.6 (10.8)		50.8 (9.2)
**Relationship to the young person, n (%)**							
	Mother	65 (76)		31 (72)		34 (79)	
	Father	13 (15)		8 (19)		5 (12)	
	Other	8 (9)		4 (9)		4 (9)	
Married or de facto married, n (%)			59 (69)		35 (81)		24 (57)
Children (n), mean (SD)			2.7 (1.6)		2.7 (1.5)		2.8 (1.6)
**Accommodation, n (%)**							
	Rented flat or room	19 (22)		8 (19)		11 (26)	
	Own flat or house	61 (71)		31 (72)		30 (70)	
	Other	6 (7)		4 (9)		2 (5)	
Born in Australia, n (%)			46 (53)		26 (60)		20 (46)
**Born outside Australia, n (%)**			40 (46)		17 (39)		23 (53)
	Oceania and Antarctica	7 (17)		3 (18)		4 (17)	
	Europe	15 (37)		6 (35)		9 (39)	
	North Africa and Middle East	2 (5)		2 (12)		0 (0)	
	Asia	5 (12)		2 (12)		3 (13)	
	Sub-Saharan Africa	11 (27)		4 (23)		7 (30)	
English main language spoken, n (%)			81 (94)		40 (93)		41 (95)
**Command of English, n (%)**							
	Good	10 (12)		6 (14)		4 (9)	
	Native speaker	76 (88)		37 (86)		39 (91)	
Highest level of education year 12, n (%)			65 (76)		31 (72)		34 (81)
**Additional qualifications, n (%)**							
	No further education	10 (12)		6 (14)		4 (9)	
	Trade or technical training	31 (36)		16 (37)		15 (36)	
	Tertiary degree	26 (31)		12 (28)		14 (33)	
	Postgraduate degree	18 (21)		9 (21)		9 (21)	
**Currently in paid work, n (%)**			69 (80)		31 (72)		38 (88)
	Full-time work	37 (54)		12 (39)		25 (66)	
**Annual income, n (%)**							
	Aus $20,799^b^ or less	16 (19)		12 (29)		4 (10)	
	Aus $28,000-51,999	23 (28)		14 (33)		9 (22)	
	Aus $52,000-77,999	20 (24)		7 (17)		13 (32)	
	Aus $78,000-103,999	11 (13)		2 (5)		9 (22)	
	Aus $104,000 or more	13 (16)		7 (17)		6 (15)	

^a^TAU: treatment as usual.

^b^At time of study commencement on October 1, 2018, the conversation rate was Aus $1=US $0.7200.

**Table 2:** Demographic characteristics of the young people receiving treatment at an early psychosis service (N=86) with Altitudes plus treatment as usual or only treatment as usual.

Variable		Total cohort (N=74)	Altitudes (n=36)	TAU^a^ (n=38)
Female (gender), n (%)		28 (38)	10 (28)	18 (47)
Age (years), mean (SD)		21.1 (3.5)	20.9 (3.8)	21.3 (3.3)
**Accommodation, n (%)**				
	House with family of origin	60 (81)	29 (81)	31 (82)
Born in Australia, n (%)		56 (76)	29 (81)	27 (71)
English main language spoken, n (%)		71 (96)	35 (97)	36 (95)
**Studying status, n (%)**				
	Not studying	45 (61)	23 (64)	22 (58)
	Studying part-time	13 (18)	8 (22)	5 (13)
	Studying full-time	16 (22)	5 (14)	11 (29)
Highest level of education year 12, n (%)		39 (53)	16 (44)	23 (62)
**Currently in paid work, n (%)**		22 (30)	13 (36)	9 (24)
	Full-time work	6 (27)	3 (23)	3 (33)

^a^TAU: treatment as usual.

### Participant Flow

**Figure 1:**
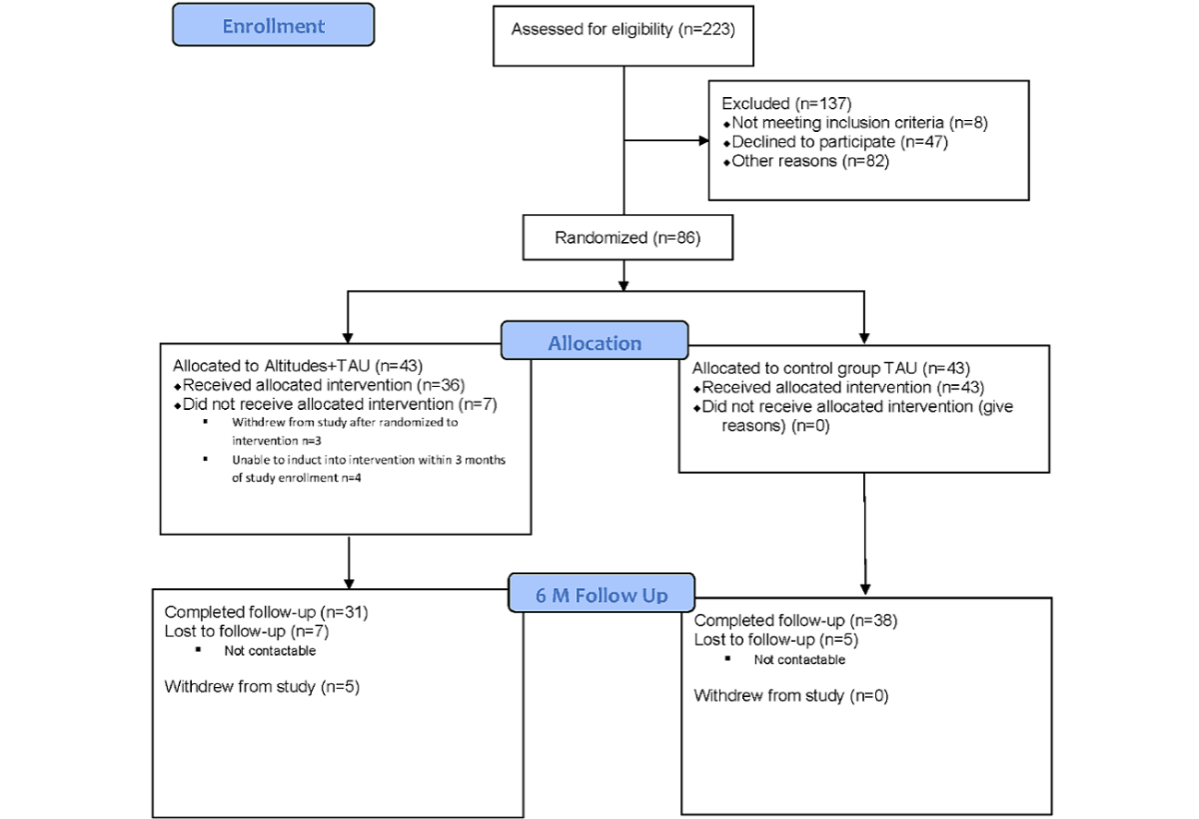
CONSORT (Consolidated Standards of Reporting Trials) flow diagram. TAU: treatment as usual.

Figure 1 shows a CONSORT diagram of the participant flow through the study. Sixty-nine caregivers had data at follow-up, indicating that missing data at follow-up were 20% (n=17). The percentage of caregivers in TAU alone that had follow-up data (n=38, 88%) did not significantly differ from caregivers in Altitudes+TAU (n=31, 72%; *χ*^2^_1_=3.6; *P*=.06). Those who did not have follow-up data were more likely to have been born in Australia (*χ*^2^_1_=4.5; *P*=.03). No other differences were found between the groups with respect to caregiver variables.

### Primary Outcome—Perceived Stress

**Figure 2:**
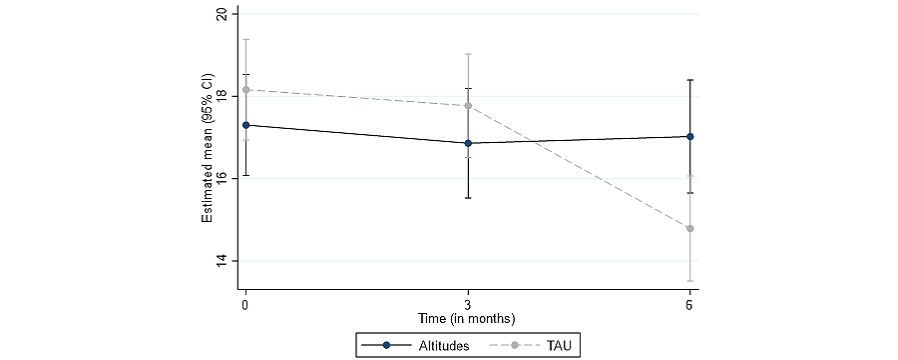
Estimated mean (95% CI) from the MMRMs for the 2 carer groups across the 3 points on the PSS. MMRMs: mixed effects repeated measures; PSS: Perceived Stress Scale; TAU: treatment as usual.

Figure 2 displays the differences between the groups with respect to PSS across baseline, at 3 and 6 months. The variance associated with family cluster was close to 0, therefore, the models were rerun with the caregiver as the unit of analysis. For the PSS, there was a significant interaction between group and time (*z*=−2.14; *P*=.03), with TAU alone showing more change (ie, improvement in PSS) from baseline to follow-up, particularly between 3 and 6 months.

### Secondary Outcomes

Table 3 comprises the adjusted means and SEs for the 2 carer groups on the secondary outcome measures. There was a significant interaction between group and time for mindfulness (*z*=2.04, *P*=.04), with the TAU alone group showing an increase over time (see Table 3). There was also a significant interaction between group and time for carer burden associated with problems with services, with the TAU alone group experiencing significantly less burden in this domain, whereas Altitudes+TAU group remained relatively stable over time (*z*=−2.10; *P*=.04; see Table 4).

With respect to changes over time regardless of group, there were significant improvements in satisfaction with life (*z*=2.02; *P*=.04; higher scores were indicative of greater satisfaction), and QoL (*z*=−2.08; *P*=.04; lower scores indicated better QoL). Both groups also improved over time with respect to emotional overinvolvement (*z*=−2.52; *P*=.01) and dependency (*z*=−2.79; *P*=.01).

**Table 3:** Estimated marginal mean (SE) for baseline and follow-up from MMRMS for secondary outcome measures for Altitudes plus treatment as usual or only treatment as usual.

Variable		Pre, mean (SE)			Post (6 months), mean (SE)			Interaction	
		Altitudes (n=43)	TAU^a^ (n=43)	Altitudes (n=31)		TAU (n=38)	Estimate, β (SE)		*P* value
**Secondary outcomes**									
	UCLA^b^ Loneliness Scale-3	25.4 (0.8)	26.9 (0.8)	26.6 (0.9)		26.3 (0.9)	−1.66 (−4.27 to 0.95)		.21
	CESD-R^c^	12.8 (1.8)	12.4 (1.8)	14.8 (2.0)		12.5 (1.9)	−1.83 (−6.32 to 2.64)		.42
	SUS^d^	59.1 (1.8)	63.2 (1.8)	61.1 (2.0)		64.1 (1.9)	−1.04 (−5.75 to 3.66)		.66
	SCS-SF^e^	3.3 (0.1)	3.3 (0.1)	3.4 (0.1)		3.6 (0.1)	.22 (−0.05 to 0.50)		.11
	SWLS^f^	19.0 (1.1)	22.1 (1.1)	21.3 (1.2)		22.6 (1.1)	−1.66 (−4.59 to 1.28)		.27
	PSWQ^g^	50.9 (2.0)	50.2 (2.0)	48.9 (2.2)		46.9 (2.0)	−1.35 (−5.85 to 3.15)		.56
	MaaP^h^	50.1 (1.1)	49.8 (1.1)	49.7 (1.3)		52.2 (1.2)	2.79 (−0.89 to 6.47)		.14
	Mindful Attention Awareness Scale	4.0 (0.1)	3.9 (0.1)	3.9 (0.2)		4.2 (0.1)	.36 (0.01 to 0.71)		.04
	MOS-SSS^i^	70.8 (2.9)	71.0 (2.9)	72.6 (3.2)		74.8 (3.0)	2.02 (−4.26 to 8.31)		.53
	MHC-SF^j^	46.2 (2.1)	45.7 (2.1)	45.6 (2.4)		46.0 (2.2)	.89 (−4.37 to 6.16)		.74
**Ways of Coping Scale**									
	Confrontive coping	0.9 (0.1)	0.9 (0.1)	1.0 (0.1)		1.0 (0.1)	−.03 (−0.30 to 0.23)		.80
	Self-controlling (sc)	1.2 (0.1)	1.4 (0.1)	1.2 (0.1)		1.3 (0.1)	−.07 (−0.34 to 0.21)		.64
	Self-controlling (ss)	1.2 (0.1)	1.3 (0.1)	1.3 (0.1)		1.2 (0.1)	−.23 (−0.61 to 0.14)		.22
	Distancing	0.8 (0.1)	1.1 (0.1)	0.8 (0.1)		0.9 (0.1)	−.13 (−0.37 to 0.10)		.26
	Escape avoidance	0.7 (0.1)	0.8 (0.1)	0.8 (0.1)		0.7 (0.1)	−.23 (−0.49 to 0.02)		.07
	Planful problem-solving	1.3 (0.1)	1.5 (0.1)	1.3 (0.1)		1.6 (0.1)	0 (−0.33 to 0.34)		.98
**Alcohol, Smoking and Substance Involvement Screening Test**									
	Tobacco	3.1 (1.1)	4.1 (1.1)	2.6 (1.2)		4.9 (1.2)	1.37 (−0.24 to 2.98)		.10
	Alcohol	7.5 (1.0)	6.5 (1.0)	7.9 (1.0)		6.4 (1.0)	−.56 (−2.68 to 1.56)		.60
	Cannabis	0.6 (0.3)	0.4 (0.3)	0.6 (0.3)		0.1 (0.3)	−.24 (−0.72 to 0.23)		.32
AQoL^k^		70.4 (1.6)	73.4 (1.6)	73.3 (1.7)		75.5 (1.6)	−.77 (−4.41 to 25.86)		.68

^a^TAU: treatment as usual.

^b^UCLA: University of California, Los Angeles, Loneliness Scale (total 20-80).

^c^CESD-R: Centre for Epidemiological Studies Depression Scale—Revised (range 0-60).

^d^SUS: Strengths Use Scale.

^e^SCS-SF: Self-Compassion Short Form (1-5).

^f^SWLS: Satisfaction With Life Scale (5-35).

^g^PSWQ: Penn State Worry Questionnaire (16-80).

^h^MaaP: Me as a Parent Questionnaire (subscales 4-20; total 16-80).

^i^MOS-SSS: Medical Outcomes Study Social Support Survey (0-100).

^j^MHC-SF: Mental Health Continuum Short Form (0-70).

^k^AQoL: Assessment of Quality of Life (0-100).

**Table 4:** Estimated marginal means (SE) for baseline and follow-up from MMRM for the Family Questionnaire and Experience of Caregiving Scale for Altitudes plus treatment as usual or only treatment as usual.

Variable		Pre, mean (SE)		Post (6 months), mean (SE)		Interaction	
		Altitudes (n=43)	TAU^a^ (n=43)	Altitudes (n=31)	TAU (n=38)	Estimate, β (SE)	*P* value
**Family questionnaire**							
	Critical comments	13.8 (1.0)	12.3 (1.0)	12.0 (1.1)	11.2 (1.1)	.68 (−1.80 to 3.16)	.59
	Emotional overinvolvement	17.5 (0.9)	17.7 (0.9)	15.5 (1.0)	15.3 (1.0)	−.45 (−2.45 to 1.54)	.66
**Experience of caregiving**							
	Difficult behaviors	14.5 (1.2)	13.7 (1.2)	13.0 (1.4)	11.1 (1.3)	−1.21 (−4.06 to 2.28)	.50
	Negative symptoms	13.5 (1.0)	14.3 (1.0)	12.4 (1.1)	12.2 (1.1)	−.93 (−3.76 to 1.91)	.52
	Stigma	7.8 (0.7)	7.8 (0.7)	6.7 (0.8)	5.4 (0.7)	−1.27 (−3.16 to 0.62)	.19
	Problem with services	10.1 (1.0)	11.6 (1.0)	9.4 (1.1)	8.2 (1.0)	−2.76 (−5.33 to −0.19)	.04
	Effects on family	11.7 (0.9)	11.7 (0.9)	11.5 (1.0)	10.6 (1.0)	−.96 (−3.25 to 1.34)	.42
	Need to back up	11.8 (0.8)	10.2 (0.8)	10.9 (0.8)	9.2 (0.8)	−.05 (−1.91 to 1.81)	.96
	Dependency	11.1 (0.7)	11.3 (0.7)	9.6 (0.8)	9.6 (0.7)	−.08 (−1.55 to 1.39)	.92
	Loss	12.0 (0.9)	13.1 (0.9)	11.2 (1.0)	11.2 (0.9)	−1.21 (−3.46 to 1.04)	.29
	Positive personal experiences	16.5 (1.0)	17.6 (1.0)	17.1 (1.1)	17.5 (1.0)	−.74 (−3.14 to 1.67)	.55
	Good aspect of the relationship	13.2 (0.7)	13.8 (0.7)	12.7 (0.7)	13.4 (0.7)	.09 (−1.70 to 1.88)	.92

^a^TAU: treatment as usual.

### Hair Cortisol

**Figure 3:**
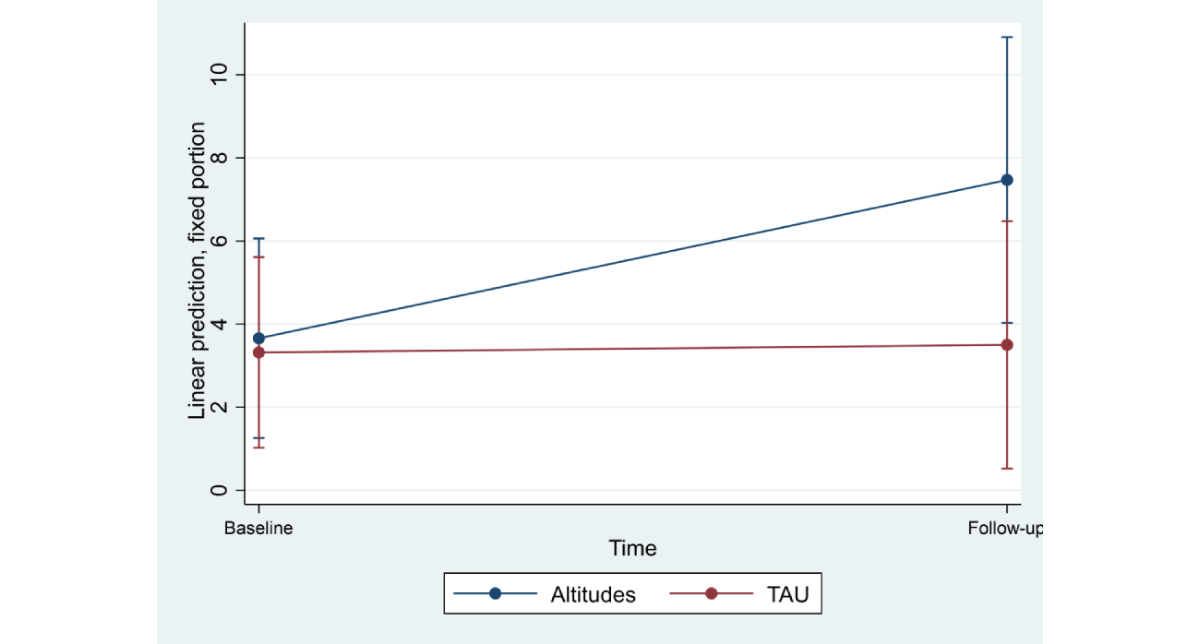
Estimated marginal mean (95% CI) for baseline and follow-up from MMRM for hair cortisol. MMRM: mixed effects repeated measure; TAU: treatment as usual.

At baseline 66 caregivers had hair cortisol data and 34 had data at follow-up. One case had very high cortisol levels with values >145 pg/mg of hair cortisol both at baseline and at follow-up. This case was an extreme outlier and distorted the estimated means, therefore was excluded from the analyses. Figure 3 details the estimated means (with 95% CI) for the hair cortisol data. The interaction between group and time was not significant (*z*=−1.46; *P*=.14); however, the time main effect was significant (*z*=2.04; *P*=.04), demonstrating a moderate increase in cortisol concentration overall and regardless of intervention. The analyses were rerun with age as a covariate, and these findings were upheld. There was no relationship between change scores on hair cortisol and the PSS (*r*=0.07; *P*=.70).

### Resource Use

As shown in Table 4 there was a significant difference between the caregiver groups with respect to early psychosis services used by the caregiver with the Altitudes group having significantly higher use of services (Fisher exact test; *P*=.01). There were no differences between the caregiver groups with respect to medication use, including use of psychotropic medications over the previous 6 months.

Data were available on resource usage for 57 young people at follow-up (see Table 5). There were no differences between groups with respect to young people’s use of emergency services and hospitalizations in the previous 6 months (see Table 5).

**Table 5:** Resource usage data in caregivers and young people.

			Altitudes, n (%)	Controls, n (%)	*P* value
**Caregivers^a^**					
	Use of early psychosis service over past 6 months		28 (93)	25 (66)	.01
	**Services from early psychosis service contact**				
		Case managers	23 (77)	22 (58)	.08
		Psychiatrists	13 (43)	13 (34)	.30
		Family peer worker (lived experience)	5 (17)	10 (26)	.26
		Family worker or carer consultant	2 (7)	5 (13)	.32
		Written psychoeducation	11 (37)	11 (29)	.34
		Community or private health services	30 (100)	36 (95)	.31
	Any medications		28 (93)	32 (84)	.22
	Psychotropic medications		10 (33)	12 (32)	.54
**Young person^b^**					
	Emergency presentation over past 6 months		6 (26)	5 (15)	.23
	Hospitalization over past 6 months		4 (17)	6 (18)	.63

^a^For Altitudes and control groups: n=30 and n=38, respectively.

^b^For Altitudes and control groups: n=23 and n=34, respectively.

### Per Protocol Analyses

There were 7 caregivers in the Altitudes+TAU treatment arm who did not receive the Altitudes intervention. Three of these cases never received the intervention or had follow-up data. One case was lost to follow-up and did not complete the intervention induction and had no follow-up data. Three cases could not be contacted, and the intervention induction was not started but they did have follow-up data. These 7 cases were excluded from per protocol analyses. There were some differences between ITT and per protocol analyses. With per protocol analyses, the interaction for PSS over the 3 time points was no longer significant (*z*=−1.73; *P=.*08); however, parental self-efficacy was significant, that is, there was a greater increase in the TAU alone group (*z*=2.01; *P*=.04). The interactions between group and time were no longer significant for mindfulness (*z*=1.86; *P*=.06) and carer burden related to problems with services (*z*=−1.63; *P*=.10).

The per protocol analyses indicated that the Altitudes+TAU group (n=22, 81%) were more likely to be in contact with the young person’s case manager than the TAU alone group (n=22, 58%; *χ*^2^_1_=4; *P*=.04).

### Altitudes Specific Measures and Outcomes

The median duration of engagement with Altitudes was 7.5 (range 1-36) weeks with median number of logins 13.5 (range 1-96); 39% (n=14) remained active for more than 11 weeks. Two participants logged on only once. The number of newsfeed posts ranged from 0 to 31 with a mean of 3.56 (SD 6.19). In relation to the carers’ perceived support for autonomy needs from web-based moderators (n=16), the mean was 5.5 (SD 1.38) on the 7-point scale. Our post hoc analysis of the relationship between activity in Altitudes and outcomes revealed that the total number of logins (*r*=0.49; *P*=.01) and the weeks of logins (*r*=0.47; *P*=.01) were both moderately and significantly correlated with improvement in PSS scores from baseline to 6 months. The number of logins (*r*=−0.46; *P*=.02) and the weeks of logins (*r*=−0.42; *P*=.03) were both moderately and negatively correlated with change from baseline to 6 months on the MOS-SSS. In relation to usability data (n=15), 80% (n=12) reported a positive and constructive experience with Altitudes and 93% (n=14) would recommend it to others.

There were no known privacy breaches or outages of Altitudes during the trial and there were no serious adverse events.

## Discussion

### Principal Results

The primary hypothesis that carers randomized to Altitudes plus family TAU would report significantly greater reductions in stress at the 6-month follow-up compared to TAU alone was not supported, with no significant change over time in self-reported severity of carer stress in the Altitudes+TAU group. The secondary hypothesis, that carers randomized to Altitudes+TAU would experience reduced hair cortisol, improved positive coping, self-efficacy, depression, and perceived social support compared with carers randomized to TAU alone at the 6-month follow-up, was also not supported. There was no improvement over 6 months on hair cortisol, mental health symptoms, loneliness, social support, self-efficacy, coping, personal strengths use, and self-compassion in the Altitudes+TAU group compared to TAU alone. There was an effect for time on stress and mindfulness with group interactions favoring the TAU alone group which was not upheld in the per protocol analysis excluding participants that did not use Altitudes. In addition, there was a group by time interaction for problems with services with TAU alone carers experiencing less burden over time which was not upheld in the per protocol analysis. There was an improvement over time, regardless of group, in satisfaction with life, QoL, emotional overinvolvement, and burden of care associated with dependency. The per-protocol analysis indicated a significant group by time interaction for parental self-efficacy with an increase for the TAU alone group.

There was no suggestion that the findings could be accounted for by the Altitudes+TAU group receiving less support outside of the intervention compared with the TAU alone group. Conversely, the Altitudes+TAU group had a higher use of services and findings from the per protocol analysis showed that the Altitudes+TAU group was more likely to be in contact with the young person’s case manager.

### Comparison With Prior Work

We have previously shown that stress appraisal improves in first-episode psychosis family caregivers through a face-to-face first-episode psychosis family intervention [55] and via bibliotherapy [56]. The current findings suggest that when added to an enhanced TAU in standard early psychosis services Altitudes did not confer benefits in relation to reducing stress, other mental health symptoms, or improving family related outcomes such as expressed emotion and perceived burden of caregiving over a the 6-month period. However, our post hoc analysis revealed significant correlations between log-ins and improvement in perceived stress and social support within the Altitudes+TAU group suggesting that with adequate engagement Altitudes may confer benefits to carers.

The stage of recovery in young patients with early psychosis may be important in the interpretation of the current findings. The greater level of contact with services and a higher level of perceived problems with services in the Altitudes+TAU group suggests that the young people in their care were experiencing more complex recoveries from the acute phase of early psychosis treatments compared to the TAU alone group. It is also possible that Altitudes led to contact with mental health services for young people. Consistent with the former interpretation, more use of Altitudes was associated with decreased stress in carers and there were no outcomes favoring TAU in our initial trial in a flagship service [20].

The median duration of engagement in Altitudes was 11 weeks (approximately 77 days) compared with 119 days in our initial trial. Given the moderate correlation between log-ins and reduced stress, it is possible that this lower level of engagement may account for the overall lack of treatment effect.

The current findings leave open the question of how the effective components of carer interventions can be translated into an accessible and effective digital version. It is probable that the mechanisms of change in effective carer interventions were not adequately targeted through Altitudes. We developed Altitudes with text- and audio-based psychoeducation; however, the format may not have adequately engaged the target skills. Educational instructional design frameworks for a digital mode may be required to ensure that continued learning outcomes are achieved [57,58]. Compared with typical behavioral family interventions [59], the duration of engagement with Altitudes was relatively brief. Continued skill development may require longer and more intensive engagement with monitoring and structured feedback on skill acquisition. Recently, bibliotherapy for early psychosis caregivers based on problem-solving, adapted for delivery via smartphone, has shown superior outcomes on carer burden, caregiving experiences, and problem-solving at the 6-month follow-up compared with either a psychoeducation family group or usual care [60]. Importantly, this intervention included repeated structured practice of problem-solving skills. Other recent findings support the incorporation of acceptance and commitment therapy principles and practice for the amelioration of burnout symptoms for early psychosis caregivers which may also have benefits for perceived stress [60].

Carers in this study overall showed improvements over time in satisfaction with life, QoL, and emotional overinvolvement. However, the trends in the proportion reporting high levels of stress and being prescribed psychotropics were notable along with increased cortisol levels potentially highlighting continued activation of the stress response system in these carers’ lives.

### Strengths and Limitations

We demonstrated that it is feasible to conduct Altitudes across sites in real-world early psychosis settings and maintain a supportive web-based environment providing highly specialized support. There were multiple measures of stress with a robust study design. The major limitation of the current study was the smaller sample size than planned, which was a consequence of a lower rate of recruitment than projected due to the unexpected complexity of recruiting carers into a research study from hub and spoke service structures. This resulted in an underpowered study which makes findings more difficult to interpret [61]. Additional limitations were the limited data regarding the young people’s mental health, which reduced the confidence in the equivalency of participant characteristics at baseline, and the exclusion of non-English speaking carer participants. Young people may have differed across groups and it was difficult to fully characterize the use of informal supports across services.

### Conclusions

This study comprised the first randomized controlled trial to investigate the effectiveness of a MOST intervention for early psychosis carers recruited from multiple real-world early psychosis programs. Altitudes was based upon our MOST which was associated with reduced emergency department visits for young people recovering from early psychosis [15]. While we did not find specific treatment effects, there were significant improvements across both treatment conditions on emotional overinvolvement, QoL, satisfaction with life, and aspects of caregiver burden and greater usage of Altitudes among carers randomized to the treatment group was associated with improvements in stress and social support. It was evident that early psychosis carers outside of flagship programs experienced levels of stress that warrant further innovations. We are currently modifying the Altitudes content to better engage mechanisms of change.

As early psychosis programs expand globally, the goal of delivering effective and accessible family interventions for carers remains an important research and clinical priority. The solid empirical foundations of effective family interventions for psychosis will continue to provide a source of inspiration for this important unmet global need.

## Appendix

Multimedia Appendix 1 Altitudes Steps.

Multimedia Appendix 2 CONSORT-EHEALTH checklist (V 1.6.1).

## Abbreviations

hEP: headspace early psychosis
HPA: hypothalamic-pituitary adrenal
ITT: intent-to-treat
MHC-SF: Mental Health Continuum Short Form
MMRM: mixed effects repeated measures
MOS-SSS: Medical Outcomes Study Social Support Survey
MOST: moderated online social therapy
PAC: Parent-Adolescent Communication
PSS: Perceived Stress Scale
PSWQ: Penn State Worry Questionnaire
QoL: quality of life
RA: research assistant
SCS-SF: Self-Compassion Short Form
SUS: Strengths Use Scale
SWLS: Satisfaction With Life Scale
TAU: treatment as usual
UCLA: University of California, Los Angeles
WHO: World Health Organization
